# The venus kinase receptor (VKR) family: structure and evolution

**DOI:** 10.1186/1471-2164-14-361

**Published:** 2013-05-30

**Authors:** Mathieu Vanderstraete, Nadège Gouignard, Arnaud Ahier, Marion Morel, Jérôme Vicogne, Colette Dissous

**Affiliations:** 1 Center for Infection and Immunity of Lille (CIIL), Inserm U1019, CNRS-UMR 8204, Institut Pasteur de Lille, 59019, Lille, France; 2 Institut de Génétique et de Biologie Moléculaire et Cellulaire (IGBMC), Inserm U964, CNRS-UMR 7104, Université de Strasbourg, 67404, Illkirch, CU Strasbourg, France; 3 UMR8161 – CNRS, Institut de Biologie de Lille, 1, rue du Pr. Calmette BP 467, 59021, Lille Cedex, France

## Abstract

**Background:**

Receptor tyrosine kinases (RTK) form a family of transmembrane proteins widely conserved in Metazoa, with key functions in cell-to-cell communication and control of multiple cellular processes. A new family of RTK named Venus Kinase Receptor (VKR) has been described in invertebrates. The VKR receptor possesses a Venus Fly Trap (VFT) extracellular module, a bilobate structure that binds small ligands to induce receptor kinase activity. VKR was shown to be highly expressed in the larval stages and gonads of several invertebrates, suggesting that it could have functions in development and/or reproduction.

**Results:**

Analysis of recent genomic data has allowed us to extend the presence of VKR to five bilaterian phyla (Platyhelminthes, Arthropoda, Annelida, Mollusca, Echinodermata) as well as to the Cnidaria phylum. The presence of NveVKR in the early-branching metazoan *Nematostella vectensis* suggested that VKR arose before the bilaterian radiation. Phylogenetic and gene structure analyses showed that the 40 receptors identified in 36 animal species grouped monophyletically, and likely evolved from a common ancestor. Multiple alignments of tyrosine kinase (TK) and VFT domains indicated their important level of conservation in all VKRs identified up to date. We showed that VKRs had inducible activity upon binding of extracellular amino-acids and molecular modeling of the VFT domain confirmed the structure of the conserved amino-acid binding site.

**Conclusions:**

This study highlights the presence of VKR in a large number of invertebrates, including primitive metazoans like cnidarians, but also its absence from nematodes and chordates. This little-known RTK family deserves to be further explored in order to determine its evolutionary origin, its possible interest for the emergence and specialization of Metazoa, and to understand its function in invertebrate development and/or reproductive biology.

## Background

Receptor Tyrosine Kinases (RTKs) are transmembrane proteins that are involved in many fundamental intra- and inter-cellular processes. RTKs have essential multicellular-specific functions, including cell-to-cell communications, control of cell proliferation and differentiation [1]. They have been found in all metazoan genomes, from the marine sponge *Geodia cydonium* to humans [2,3]. Moreover, RTKs were also shown to be present in choanoflagellates [4,5] and Filasterea [6], which are the sister groups of Metazoa. A large number of RTKs are conserved throughout evolution, but unique and organism-specific RTKs have been identified, such as *Sweet tooth* in *Hydra vulgaris*[7] or *kin15*/*kin16* in *Caenorhabditis elegans*[8,9]. RTKs have been classified into distinct families, depending on the modular composition of their extracellular domains and their ability to bind different types of ligands, as well as by their kinase domain sequences. The human genome encodes 58 RTKs, and these receptors are classified into 20 families [10]. According to the data regrouped in http://kinase.com, the invertebrate model organisms *Drosophila melanogaster* and *C*. *elegans* possess 16 and 29 RTKs and share 11 and 10 families with human RTKs, respectively [3].

Venus Kinase receptors (VKRs) constitute an RTK family, originally found in the parasite platyhelminth *Schistosoma mansoni*[11], then in several other invertebrates (insects and echinoderms). However, no VKR could be found in any chordate genome and, more strikingly, this receptor was not present in *C*. *elegans* and *D*. *melanogaster*[12]. VKR proteins possess an atypical structure [11,12], containing an intracellular tyrosine kinase (TK) domain similar to that of insulin receptors, and an extracellular Venus Flytrap (VFT) domain. VFTs were first identified as bacterial periplasmic-binding proteins involved in the transport of small molecules, such as amino acids, sugars or ions and they constitute the binding pocket of different receptor types such as class C G-protein coupled receptors [13]. The VFT domain of VKR is related to that of the ANF receptor in protein family databases (pfam 01094) [12]. VKR kinases can be activated following the binding of amino-acids to the extracellular VFT domain [12,14], and this opens interesting perspectives on a novel mechanism for RTK activation as well as on possible specific and new functions of these receptors in cellular signalling.

In this paper, we present an up-dated version of the VKR family, resulting from an exhaustive research of VKR orthologs in the published genomes of a variety of organisms belonging to major phyla in Metazoa. We show that *vkr* genes are present at least in five major phyla in Bilateria (Platyhelminthes, Arthropoda, Annelida, Mollusca, Echinodermata), and also, more strikingly, in the cnidarian *Nematostella vectensis*. Phylogenetic analyses indicated that all the putative protein sequences grouped monophyletically, and a new version of the VKR phylogeny was built. *In silico* analyses and multiple alignments of the VFT and TK functional domains of VKRs allowed us to reinforce the structural model of the receptor and to get a better prediction of potential ligands and kinase activity of VKRs.

## Results and discussion

### A large distribution of *vkr* genes in eumetazoan genomes

Previous studies have shown that VKRs constitute a distinct RTK family. These receptors were described in 15 different protostomes, including insects and the platyhelminth *S*. *mansoni*. A *vkr* gene was also found in the deuterostome *Strongylocentrotus purpuratus*[12]. The aim of this study was to extend the VKR family to a large number of phyla in order to evaluate the place of these receptors in the animal kingdom and to contribute to a better understanding of their evolution.

**Figure 1:**
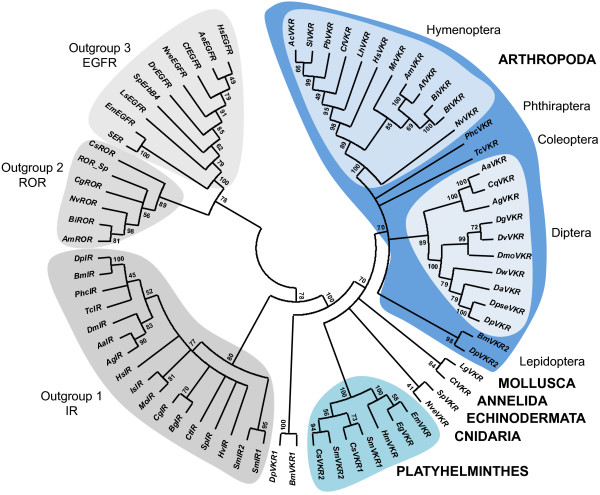
**Phylogenetic construction of the VKR family.** A maximum likelihood tree was generated from the 40 VKR sequences using MEGA5 under the JTT+G+I model with 100 bootstrap repetitions. Outgroups are formed by insulin receptors (IR, outgroup 1), RTK-like orphan receptors (ROR, outgroup 2) and/or EGF receptors (EGFR, outgroup 3) of the following species: *A*. *aegypti* (AaIR, AAB17094.1), *A*. *echiniator* (AeEGFR, EGI67610.1), *A*. *gambiae* (AgIR, EAA00322.3), *A*. *mellifera* (AmROR, XP_397058.4), *B*. *impatiens* (BiROR, XP_003490221.1 ), *Biomphalaria glabrata* (BgIR, AAF31166.1), *B*. *mori* (BmIR, NP_001037011.1), *C*. *floridanus* (CfEGFR, EFN60989.1), *Crassostrea gigas* (CgIR, EKC21734.1; CgROR, EKC27495.1), *C*. *sinensis* (CsROR, GAA34401.2), *C*. *teleta* (CtIR, ELT96360.1), *D*. *melanogaster* (DmIR, AAC47458.1), *D*. *plexippus* (DplIR, EHJ65074.1), *D*. *virilis* (DvEgfr, ABD64816.1), *E*. *multilocularis* (EmEGFR, CAD56486.1), *H*. *saltator* (HsIR, EFN83767.1; HsEGFR, EFN75184.1) *Hydra vulgaris* (HvIR, Q25197.1), *Ixodes scapularis* (IsIR, XP_002416224.1), *Lymnaea stagnalis* (LsEGFR, ABQ10634.1), *Metaseiulus occidentalis* (MoIR, XP_003739590.1), *N*. *vitripennis* (NvROR, XP_001601308.2; NvEGFR, XP_001602830.2), *P*. *humanus corporis* (PhcIR, XP_002430961.1), *S*. *mansoni* (SmIR1, GenBank: AAN39120.1; SmIR2, GenBank: AAV65745.2 and SER, GenBank: AAA29866.1), *S*. *purpuratus* (SpIR, XP_784376.3; SpErbB4, XP_791361.3 and ROR_Sp, XP_003729469.1) and *T*. *castaneum* (TcIR, EFA11583.1). For VKR abbreviations and accession numbers, see Table 1.

**Figure 2:**
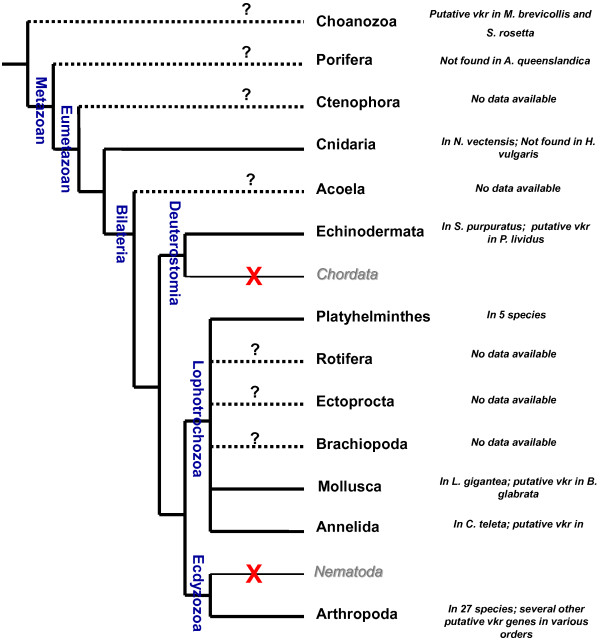
**Distribution of vkr genes in metazoan phyla.***Vkr* genes were definitely identified in Arthropoda (27 insect species), Platyhelminthes (trematode and cestodes parasites), and in Mollusca, Annelida and Echinodermata (at least in one species for each phylum). Additionally, putative VKR sequences have been detected in the genomes of many other arthropods, in the mollusc *Biomphalaria glabrata*, in the annelid *Helobdella robusta* and in the echinoderm *Paracentrotus lividus*. The presence of VKR has been confirmed in the cnidarian *N*. *vectensis* but not found in Hydra (*H*. *magnipapillata* and *H*. *vulgaris*) genomes. The “unclassified” TK (UTK12) [5] and the RTKS kinase [17] found respectively in the choanoflagellates *Monosiga brevicollis* and *Salpingoeca rosetta* possess an architecture similar to VKR proteins. However, no *vkr* gene was found in the poriferan *Amphimedon queenslandica*. Concerning Ctenophora, Acoela, Rotifera, Ectoprocta, Brachiopoda phyla, genomic data are currently not sufficient to assess the presence or not of *vkr* genes in these organisms. No *vkr* genes were found in Chordata (vertebrates) nor in Nematoda (worms).

Using BLAST approaches, we searched for *vkr* genes in multiple genomic databases (see Methods). Results showed that *vkr* genes were present in at least 36 species from six distinct phyla (Cnidaria, Echinodermata, Platyhelminthes, Mollusca, Annelida and Arthropoda, see Table 1). The protein architecture of each putatively encoded VKR was analysed using the SMART online software http://smart.embl-heidelberg.de/ in order to verify that newly discovered genes effectively encoded structurally conserved proteins, composed of an extracellular VFT module and an intracellular TK domain linked together by a single transmembrane α-helix. In Platyhelminthes, two putative *vkr* genes were detected in both *Clonorchis sinensis* and *S*. *mansoni*[14] parasitic trematodes. By contrast, only one *vkr* was found in the genome of the three parasitic cestodes, *Echinococcus multilocularis*, *Echinococcus granulosus* and *Hymenolepis microstoma*. No unequivocal VKR sequence could be found in the genome of the planarian *Schmidtea mediterranea*, but a sequence encoding a polypeptide composed of a TK domain and a truncated VFT module (GenBank: AAWT01078636.1) was identified, suggesting that a *vkr* gene might still exist in Turbellaria. However, deeper investigations are needed to extend reliably the existence of VKR to planarian species and to the whole of the Platyhelminth phylum. Several new *vkr* genes were also found in diverse insect genomes. We identified 15 new *vkr* genes in 13 species belonging to the Hymenoptera and Lepidoptera orders. In Hymenoptera, on top of the two genes previously characterized in the honeybee *Apis mellifera* and in the parasitic wasp *Nasonia vitripennis*[12], we were able to identify 11 new *vkr* genes in the genomes of eight Formicidae and three Apidae species. In Lepidoptera, we could show that both the silkworm *Bombyx mori* and the monarch butterfly *Danaus plexippus* possess two *vkr* genes located in tandem on a single scaffold (GenBank accession numbers DF090406.1and JH386161.1, respectively). Additionally, we could find truncated *vkr* sequences in other arthropod genomes, such as those of *Rhodnius prolixus* (Hemiptera), *Glossina morsitans morsitans* (Diptera) and *Daphnia pulex* (Cladocera). However, the bad scores of similarity registered from sequence alignments and the lack of protein structure conservation led us to exclude these sequences from further phylogenetic analyses. These were performed to assess that the predicted proteins were VKR orthologs belonging to the same family. A maximum likelihood phylogenetic tree was generated under the JTT+I+G model with the support of three outgroups composed respectively of insulin (outgroup 1), RTK-like orphan (ROR) (outgroup 2) and epidermal growth factor (EGF) (outgroup 3) receptors of invertebrates (Figure 1). Results showed that all VKR sequences consistently form a monophyletic group distinct from the three other RTK families. Inside of the VKR family, distinct and robust groups were formed by hymenopteran, coleopteran or dipteran sequences. Platyhelminth VKRs form a cluster made of two distinct cestode and trematode branches. In the trematode branch, VKR1 proteins grouped distinctly from the VKR2 ones. Similarly, lepidopteran VKR1 and VKR2 sequences (BmVKR1/2 and DpVKR1/2) were split into two different branches, but surprisingly lepidopteran VKR1 proteins were rejected out of the arthropod group and placed at the root of the VKR tree. Finally, the cnidarian (NveVKR), annelid (CtVKR), mollusc (LgVKR) and echinoderm (SpVKR) proteins were correctly bound to the VKR tree, but no conclusion can be drawn about their phylogenetic proximity since only one *vkr* is available for each phylum. Thus, in these studies, we have confirmed the distribution of VKR in arthropods (in insects particularly), platyhelminths and echinoderms and found new VKR orthologs in three additional phyla, Annelida (*Capitella teleta*), Mollusca (*Lottia gigantea*) and Cnidaria (*N*. *vectensis*) (Figure 2). Additionally, putative VKR sequences were found in EST databases of different orders of Arthropoda (*Cochliomyia hominivorax* (gb|FG295125.1), *Rhipicephalus microplus* (gb|FG302900.1), *Coptotermes formosanus* (GI:345171826) and *Crassostrea gigas* (GI:313329111)), in the mollusc *Biomphalaria glabrata* (Contig1132.1, *Biomphalaria glabrata* Genome Initiative, biology.unm.edu/biomphalaria-genome/index.html), in the annelid *Helobdella robusta* (gb|EY370614.1) and in the echinoderm *Paracentrotus lividus* ( emb|AM524433.1). The identification of a putative VKR in this second echinoderm indicates that *vkr* genes could be present in multiple deuterostomes, and excludes a recent horizontal gene transfer or a genomic material contamination. The presence of VKR in Cnidaria, an animal lineage early diverging from Bilateria, suggests that the VKR family emerged prior to the expansion of Bilateria, the clade that comprises almost all extant animals. However, though we identified NveVKR in the anthozoan *Nematostella*, we could not detect any VKR sequence in the genome of the hydrozoan *Hydra magnipapillata*, a result in agreement with the recent genome-wide RTK screening performed for this species [15]. Such a difference could be related to those already described for gene diversity and content between anthozoan and hydrozoan genomes [16]. At this time, cnidarians are the first branch of Metazoa in which *vkr* genes have been found and the question of their presence or not throughout all phyla of Bilateria is still open. Extensive research in vertebrate and roundworm genomes allowed us to conclude that VKR is absent from Chordata and Nematoda but the lack of genomic data for species of the Acoela, Rotifera, Ectoprocta and Brachiopoda phyla still stands in the way of a better understanding of the place of VKR throughout evolution (Figure 2). Moreover, the existence of *vkr* in Premetazoa remains to be considered because we have recently noticed in choanoflagellates that the “unclassified” TK annotated as UTK12 of *Monosiga brevicollis*[5] as well as the RTKS kinase of *Salpingoeca rosetta*[17] have both a protein architecture similar to that of VKR proteins.

**Table 1:** **Complete list of the 40 *vkr *genes found in genomic databases**

**Species**	**Class**	**Name**	**Accession number**	**Database**
*Acromyrmex echiniator*	Insect	AeVKR	GL888498.1	http://flybase.org/
*Aedes aegypti*	Insect	AaVKR	DAA06509.1	http://blast.ncbi.nlm.nih.gov/Blast.cgi
*Anopheles gambiae*	Insect	AgVKR	ACF34410.1	http://blast.ncbi.nlm.nih.gov/Blast.cgi
*Apis florea*	Insect	AfVKR	GL576580.1	http://flybase.org/
*Apis mellifera*	Insect	AmVKR	ACF34409.1	http://blast.ncbi.nlm.nih.gov/Blast.cgi
*Atta cephalotes*	Insect	AcVKR	GL377380.1	http://flybase.org/
*Bombus impatiens*	Insect	BiVKR	XP_003486761.1	http://ncbi.nlm.nih.gov/
*Bombus terrestris*	Insect	BtVKR	GL898830.1	http://flybase.org/
*Bombyx mori*	Insect	BmVKR1	DF090406.1	http://flybase.org/
*Bombyx mori*	Insect	BmVKR2	DF090406.1	http://flybase.org/
*Camponotus floridanus*	Insect	CfVKR	EFN73169.1	http://ncbi.nlm.nih.gov/
*Capitella teleta*	Annelid	CtVKR	136189	http://genome.jgi.doe.gov/pages/search-for-genes.jsf?organism=Capca1
*Clonorchis sinensis*	Trematode	CsVKR1	GAA27163.2	http://ncbi.nlm.nih.gov/
*Clonorchis sinensis*	Trematode	CsVKR2	GAA49307.1	http://ncbi.nlm.nih.gov/
*Culex quinquefasciatus*	Insect	CqVKR	DAA06510.1	http://ncbi.nlm.nih.gov/
*Danaus plexippus*	Insect	DplVKR1	EHJ69301.1	http://ncbi.nlm.nih.gov/
*Drosophila ananassae*	Insect	DaVKR	DAA06508.1	http://ncbi.nlm.nih.gov/
*Drosophila grimshawi*	Insect	DgVKR	DAA06505.1	http://ncbi.nlm.nih.gov/
*Drosophila mojavensis*	Insect	DmoVKR	DAA06504.1	http://ncbi.nlm.nih.gov/
*Drosophila persimilis*	Insect	DpVKR	DAA06507.1	http://ncbi.nlm.nih.gov/
*Drosophila pseudoopscura*	Insect	DpseVKR	ACF34407.1	http://ncbi.nlm.nih.gov/
*Drosophila virilis*	Insect	DvVKR	DAA06503.1	http://ncbi.nlm.nih.gov/
*Drosophila wilistoni*	Insect	DwVKR	DAA06506.1	http://ncbi.nlm.nih.gov/
*Echinococcus granulosus*	Cestode	EgVKR	NODE_166072	http://www.sanger.ac.uk/resour…s/echinococcus-granulosus.html
*Echinococcus multilocularis*	Cestode	EmVKR	pathogen_EMU_scaffold_007728	http://www.sanger.ac.uk/resour…s/echinococcus-multilocularis.html
*Harpegnathos saltator*	Insect	HsVKR	EFN85558.1	http://ncbi.nlm.nih.gov/
*Hymenolepis microstoma*	Cestode	HmVKR	744	http://www.sanger.ac.uk/resour…hs/hymenolepis-microstoma.html
*Linepithema humile*	Insect	LhVKR	GL905323.1	http://flybase.org/
*Lottia gigantea*	Mollusc	LgVKR	109151	http://genome.jgi-psf.org/pages/search-for-genes.jsf?organism=Lotgi1
*Megachile rotundata*	Insect	MrVKR	GL985818.1	http://flybase.org/
*Nasonia vitripennis*	Insect	NvVKR	DAA06502.1	http://ncbi.nlm.nih.gov/
*Nematostella vectensis*	Anthozoan	NveVKR	SB_43850	http://nematostella.bu.edu/stellabase/
*Pediculus humanus corporis*	Insect	PhcVKR	DAA06501.1	http://ncbi.nlm.nih.gov/
*Pogonomyrmex barbatus*	Insect	PbVKR	GL738256.1	http://flybase.org/
*Schistosoma mansoni*	Trematode	SmVKR1	AAL67949.1	http://ncbi.nlm.nih.gov/
*Schistosoma mansoni*	Trematode	SmVKR2	ADD91576.1	http://ncbi.nlm.nih.gov/
*Solenopsis invicta*	Insect	SiVKR	EFZ12829.1	http://ncbi.nlm.nih.gov/
*Strongylocentrotus purpuratus*	Echinoidea	SpVKR	DAA06500	http://ncbi.nlm.nih.gov/
*Tribolium castaneum*	Insect	TcVKR	ACF34408.1	http://ncbi.nlm.nih.gov/

*Vkr* gene name is specified for each species, as well as protein or scaffold accession number and website link of the database in which the gene was found.

### Diversification of *vkr* genes

Previous studies have already demonstrated that *vkr* genes were substantially heterogeneous in length as well as in intron-exon composition throughout species, while their organization within a given order was rather well conserved, like in Diptera [12]. In this work, we have made an extensive study of the exon/intron structure of all the discovered *vkr* genes using the GenScan [18] and Augustus [19] gene prediction Web servers. Data in Table 2 illustrate the gene composition of *vkr* genes from all the species grouped into families. They indicate for each *vkr* gene the total number of exons and specify the exons that encode VFT, TM or TK domains. The results confirmed a wide heterogeneity for *vkr* genes across the diverse phyla. In arthropods, *vkr* genes have highly variable size (estimated from 4 to 65 kb) and exon numbers (from 5 in Drosophilidae and Culicidae to 13 in the ant *Acromyrmex echiniator*). As it was already observed in the case of dipterans [12], sizes and/or exon-intron structures were homogeneous inside of the hymenopteran order, particularly for Formicidae and Apidae. The low number of exons found in *vkr* genes of Drosophilidae and Culicidae could be due to the high degree of intron loss that has occurred during the evolution of the protostome lineage leading to flies and mosquitoes [20].

**Table 2:** **Characteristics and structural organization of *vkr *genes**

**Origin**	**Name**	**Size**	**Exon number**	**Coding exons for :**		
				**VFT**	**TM**	**TK**
**Cnidaria**						
Anthozoa						
Edwardsiidae						
	Nvevkr	13,5 kb	15	E2-E7	E12	E12-E15
**Arthropoda**						
Diptera						
Drosophilidae						
	Dvvkr	3,8 kb	5	E2-E5	E5	E5
★	Dpseuvkr	3,8 kb	5	E2-E5	E5	E5
	Dmovkr	3,8 kb	5	E2-E5	E5	E5
	Davkr	3,8 kb	5	E1-E4	E4	E5
	Dpvkr	3,8 kb	5	E2-E5	E5	E5
	Dwvkr	3,8 kb	5	E2-E5	E5	E5
	Dgvkr	3,8 kb	5	E2-E5	E5	E5
Culicidae						
	Cqvkr	18 kb	5	E2-E5	E5	E5
	Aavkr	48 kb	5	E2-E5	E5	E5
Anophilinae						
★	Agvkr	65kb	9	E6-E9	E9	E9
Hymenoptera						
Formicidae						
	Pbvkr	6,5 kb	11	E4-E8	E8	E9-E11
	Sivkr	5,5 kb	9	E4-E6	E8	E8-E9
	Hsvkr	7 kb	11	E4-E7	E7	E8-E11
	Acvkr	7 kb	11	E4-E6	E6	E7-E11
	Lhvkr	6,5 kb	11	E3-E5	E5	E6-E7
	Cfvkr	8 kb	11	E4-E8	E8	E8-E9
	Aevkr	15,5 kb	13	E3-E6	E6	E7-E10
Apidae						
	Afvkr	8,5 kb	12	E5-E8	E9	E9-E12
★	Amvkr	8 kb	11			
	Bivkr	37 kb	12	E4-E7	E7	E8-E12
	Btvkr	37 kb	12			
Megachilidae						
	Mrvkr	5 kb	8	E2-E5	E5	E6-E8
Pteromalidae						
	Nvvkr	27 kb	12	E6-E11	E12	E12
Lepidoptera						
Bombycidae						
	Bmvkr1	24 kb	5	E5	E5	E5
	Bmvkr2	7 kb	5	E2-E4	E4	E4
Nymphalidae						
	Dplvkr1	7 kb	6	E4-E5	E5	E5-E6
	Dplvkr2	21,5 kb	8	E3-E7	E7	E7-E8
Coleoptera						
Tenebrionidae						
★	Tcvkr	16 kb	5	E2-E4	E4	E4-E5
Phthiraptera						
Pediculidae						
	Phcvkr	9,5 kb	10	E4-E8	E8	E8-E10
**Annelida**						
Polychaeta						
Capitellidae						
	Ctvkr	8,5 kb	18	E4-E11	E12	E12
**Mollusca**						
Gasteropoda						
Lottiidae						
	Lgvkr	9,5 kb	17	E4-E10	E11	E11-E15
**Platyhelminths**						
Trematoda						
Schistosomatidae						
★	Smvkr1	30 kb	16	E7-E10	E10	E11-E14
★	Smvkr2	30 kb	18	E7-E10	E10	E11-E14
Opistorchiidae						
	Csvkr1	48 kb	15	E5-E9	E9	E10
	Csvkr2	34 kb	15	E4-E8	E8	E8-E11
Cestoda						
Taeniidae						
	Egvkr	23,5 kb	15	E3-E7	E9	E10-E12
	Emvkr	19 kb	16	E4-E8	E10	E11-E13
Hymenolepididae						
	Hmvkr	19 kb	11	E1-E6	E7	E8-E10
**Echinodermata**						
Echinoida						
Strongylocentrotidae						
★	Spvkr	60 kb	21	E9-E14	E16	E17-E21

Data illustrate the composition *of vkr* genes from all the animal species (see Table 1) which have been classified into their respective phylum, order and family. They indicate for each *vkr* gene the number of exons and specify those that encode VFT, TM or TK domains. Stars indicate genes for which the complete cDNA sequence was obtained.

*Vkr* genes found in lophotrochozoan organisms (annelids, molluscs, platyhelminths), are overall more complex (15 to 18 exons) than the insect ones, except in the cestode *H*. *microstoma* (11 exons). Lophotrochozoan *vkr* genes are according to this more similar to that one detected in the phylogenetically basal animal *N*. *vectensis*. Indeed, *Nvevkr* is also intron-rich (15 exons), respecting therefore the known high complexity of the genes present in early animal genomes [20]. *Spvkr* found in the echinoderm *S*. *purpuratus* remains the most complex *vkr* gene found with a size of 60kb and a total of 21 exons. In trematodes, the organisation of *Smvkr1* and *Smvkr2* genes was shown to be quite identical (see Table 2, and [14]), arguing for a duplication event in *S*. *mansoni*.

**Figure 3:**
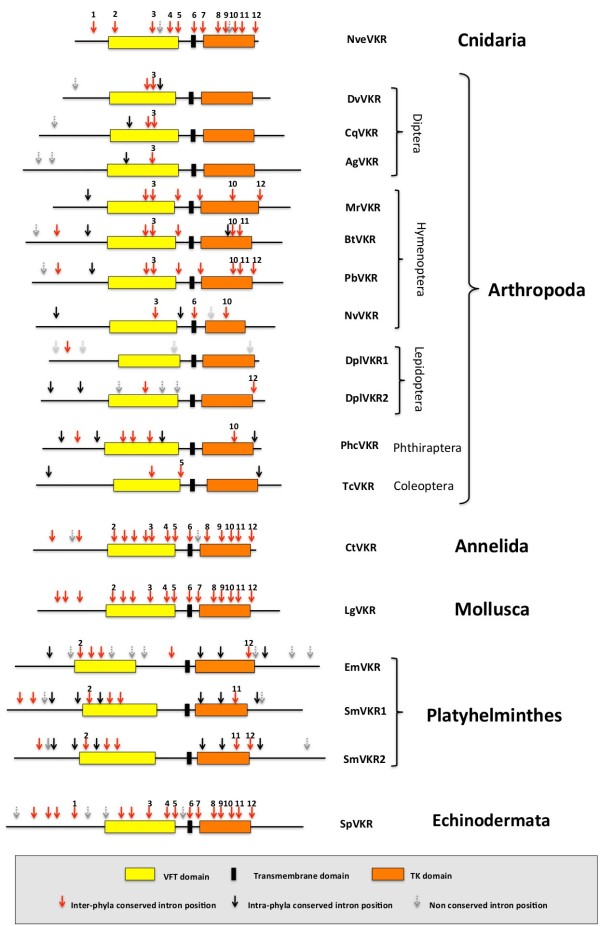
**Schematic representation of the organisation of *vkr *genes.** Coding sequences of genes selected in the different groups of organisms are represented. Boxes indicate respectively the positions of VFT, TM or TK domains. For each gene, arrows indicate the presence of introns at conserved positions either in several phyla (red arrows) or inside of a given phylum (black arrows). Grey arrows indicate the presence of introns at non conserved positions.

As a preliminary approach to understand *vkr* gene evolution, we have analysed the conservation of intron positions throughout the diverse animal groups (Figure 3). First results overall indicated that *vkr* genes were all sharing at least one conserved intron position, arguing that they might belong to the same family.

We also noticed that 12 out of 14 intron positions of *Nvevkr* were found in at least one phylum and that most of these conserved exon-intron boundaries were located in regions that encode VFT and TK domains. Most of these positions were shown to be conserved in the annelid (*Ctvkr*), mollusc (*Lgvkr*) and echinoderm (*Spvkr*) genes. Inversely, only a very limited number of intron positions seem to be conserved in arthropods as well as in platyhelminthes, indicating a profound reorganization of *vkr* genes along evolution. Finally, specific or non-conserved intron positions were also found in various *vkr* genes (Figure 3). Taken together, these studies demonstrate that *vkr* genes are highly variable in size and in complexity but that, in spite of their heterogeneity, all of them possess common features, which are conserved from Cnidaria to the other phyla.

### Conservation of VKR tyrosine kinase domains

Using the multiple alignment ClustalW algorithm, we have compared the TK domain sequences of the 40 VKRs and generated an identity matrix. As it could be expected for catalytic structures, the sequences are relatively well conserved across all species, with the best scores of identity observed between the species belonging to a same order. For example, in Hymenoptera the TK domains of *Bombus* and *Apis* species are more than 92% identical, in Diptera, those of *Drosophila* species are more than 75% identical and those of the mosquitoes *A*. *gambiae* and *A*. *aegypti* are 93%. For platyhelminth VKRs, identities between TK domains scored between 57 to 96%, with the best score registered between the two cestode parasites *E*. *multilocularis* and *E*. *granulosus*. In Lepidoptera, the TK domains of *Bombyx* and *Danaus* VKRs were less conserved, except for BmVKR2 and DplVKR2 that share 81% of identity.

**Figure 4:**
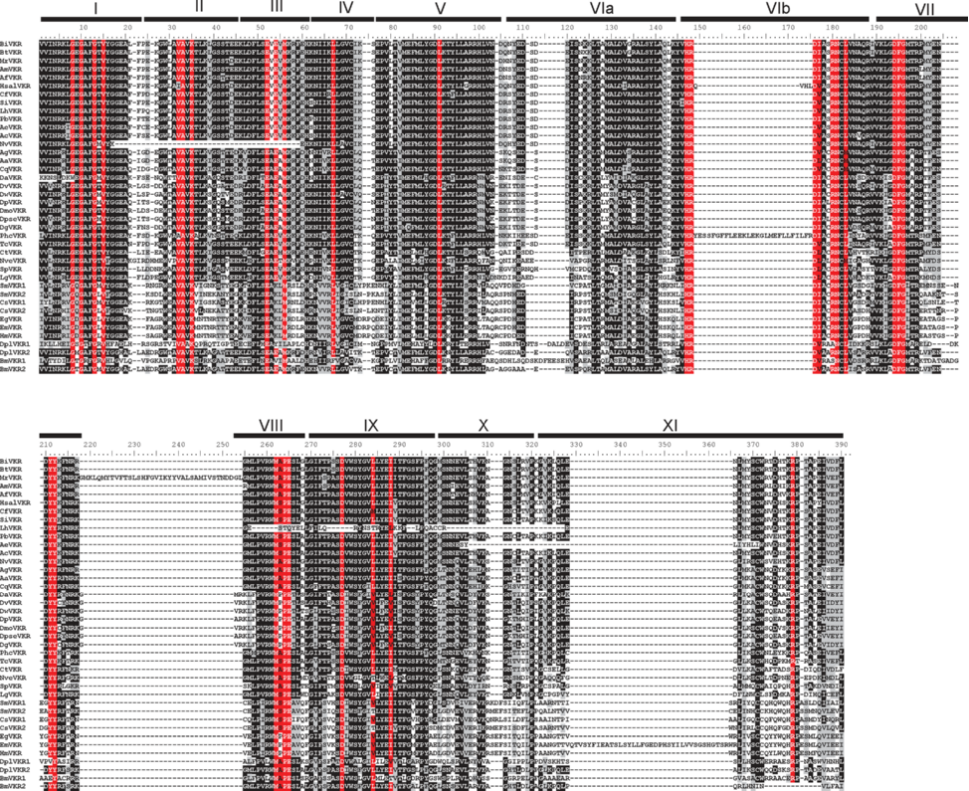
**Alignment of the TK sequence of VKR proteins using the CLUSTALW algorithm.** Numbers I to XI correspond to the eleven subdomains conserved in protein kinase domains. Essential motifs for protein kinase activity are indicated in red. The motif G_8_xGxxGxV_15_ is required for binding of ATP and V_32_AxK (16×)E_52_ for its stabilization. The motif H_147_RD(L/V/I)xxRNxL_156_ is implicated in phosphotransfer and the triplet D_194_FG_196_ is the Mg^2+^ binding site. The two residues Y_211_ and Y_212_ constitute the autophosphorylation site and the motif M_262_(A/S)PE_265_ is required for stabilization of the active kinase core.

In the aligned sequences, we could identify most of the residues shown to be essential for TK activity [21]. As indicated in Figure 4, the glycine-rich motif G_8_xGxxGxV_15_ required for the correct positioning of ATP is found in all VKRs except in that of *D*. *ananassae*, which lacks the first G residue. The V_32_AxK(16x)E_52_ motif essential for ATP stabilization is also tightly conserved, except for the hymenopteran *N*. *vitripennis* VKR. The phosphotransfer site H_147_RD (L/V/I)xxRNxL_156_ is also present in the catalytic loop of all VKRs but in the insect VKRs of *P*. *humanus corporis* and *H*. *saltator*, this motif is interrupted by an insertion. Without exception, all VKRs possess the D_194_FG_196_ site essential for the binding to the catalytic magnesium ion. In the activation loop, the two juxtaposed Y_211_Y_212_ autophosphorylation site that allows an open access to ATP and substrates in many activated RTKs (like insulin receptors) [22], is found in all VKRs, except in VKR1 isoforms of *B*. *mori* and *D*. *plexippus* in which a single Y is found at this position. The M_262_(A/S)PE_265_ motif implicated in the stabilization of the active kinase core is present in most VKRs, but the A/S residue is replaced by a P residue in all the *Drosophila* species as well as in cestodes. We can note also that this motif is totally absent from the VKR of the ant *L*. *humile*. Other hydrophobic residues M_56_, L_67_, L_91,_ (L/V/I)_284,_ (L/V/I)_288_ , that compose in every active kinase spatially conserved motifs, termed spines, and that play a major role in kinase dynamic assembly and activity [23], are present in all VKRs. Overall, these data showed that a large part of VKR sequences contained all the motifs essential for TK activity. Furthermore, our previous demonstrations [12,14] that recombinant receptors from *A*. *mellifera* and *S*. *mansoni* were catalytically active and able to autophosphorylate, strongly suggest that most of the VKRs identified in this work might exhibit similarly kinase activity. Concerning the ones (DaVKR, NvVKR, BmVKR2, DplVKR2, PhcVKR, HsVKR and LhVKR), that lack one or several motifs essential for catalytic activity, we could suggest that they constitute dead or pseudokinases, but this conclusion should be taken with much caution because of possible artefacts in gene prediction or sequences. Further analyses of their kinase potential would be needed to conclude.

### Conservation and divergence of VKR ligand-binding domains

**Figure 5:**
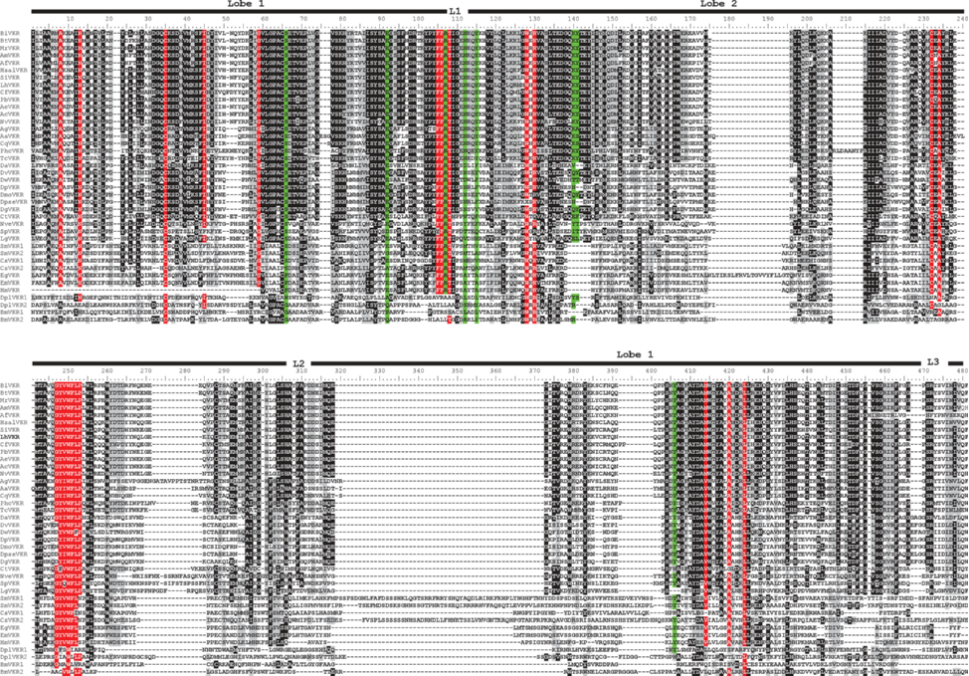
**Alignment of the VFT sequence of VKR proteins using the CLUSTAL W algorithm.** Lobe I and Lobe II (indicated by the upper black line) and the three linkers (L1, L2, L3) constitute the structure of VFT domains. Residues highlighted in red are highly conserved in all or most VKR sequences, and residues highlighted in green correspond to the consensus amino-acid binding motif of VFT domains [24].

Multiple alignment of the VFT module sequences from the 40 VKRs shows that protein sequences are relatively well conserved, particularly in insect species (Figure 5). Excluding the two lepidopteran VKRs, all insect VFT sequences share from 29% (between PhcVKR and DpseVKR) to 98% (between the two *Bombus* species) of identity. Interestingly, VFT sequences are highly conserved within a given order. As an example, VKRs from the 13 hymenopteran species all share at least 70% identity. Likewise, there is at least 50% identity between the VKRs identified in all dipteran species. Taken together, these results agree with an evolutionary conservation of insect VFT sequences. However, an exception concerns the lepidopteran VKRs (BmVKR1/2 and DplVKR1/2) for which VFT module sequences are highly divergent from each other and from all insect VFT domains (less than 16% of identity). Still, the BmVKR2 VFT module seems closer to that of DplVKR2 (40% of identity). Concerning the VFT sequences of platyhelminths, intermediate levels of conservation were observed between trematode and cestode sequences (24% to 44% identity) while we could note, as previously observed for the TK domain, a marked identity (93%) within the genus *Echinococcus* between EgVKR and EmVKR VFT domains. Finally, other VKRs (from Annelida, Mollusca and Cnidaria species) were found to be highly divergent from platyhelminth and lepidopteran VKRs in their VFT domains, but as discussed above concerning their phylogeny (Figure 1), available sequences are not sufficient to state about receptor evolution in these phyla.

**Figure 6:**
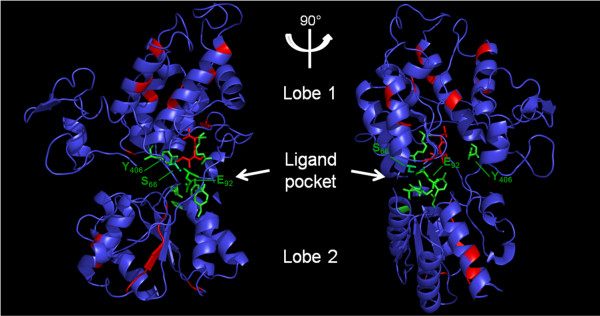
**Evolutionary conservation of residues of the VFT domain visualized on the comparative modeling of AmVKR based on mGluR5 crystal (PBP: 3lmkA).** Structurally important motifs are indicated in red and residues composing the consensus amino-acid binding motif of VFT domains [24] are in green sticks (for details see Figure 5). The highly conserved residues S_66_, E_92_ and Y_406_, found to be important for amino-acid binding, are localized within the binding pocket, together with two conserved structural residues T_108_ and I_109_ shown in red sticks.

VFT modules constitute the binding pocket of various receptors activated by small molecules [13]. They are composed of two lobes connected *via* flexible tethers that close around the bound ligand. In most class C GPCRs, these modules contain the binding site for natural amino acids or derivatives and ligand recognition is dependent on a consensus motif of 8 residues that participate to the binding of the α-amino acid functions (i.e. primary amine and carboxylic acid) [24]. Among these residues, the serine that binds the COOH group of glutamate in mGluR1 (Ser_165_), is the most conserved residue in class C GPCRs. In most VKRs (except in cestode and lepidopteran receptors), this residue is strictly conserved (at position 66 in the VFT sequence, Figure 5). The R_107,_ another important residue composing the consensus site for AA binding in VFT modules [24] is also present in all VKRs (except in Lepidoptera). These observations are totally in agreement with our demonstration that amino-acids, and particularly L-arginine, are able to bind and activate both honey bee and schistosome VKRs [12,14] and we have recently confirmed the requirement of S_66_ and R_107_ for the amino-acid recognition by VKR receptors (unpublished). Interestingly, if the six other elements composing the consensus motif in VFT modules for amino acid binding [24] are different in the ligand-binding domains of VKRs, we could note that at their exact positions, many VFT domains present identical residues (highlighted in green in Figure 5), and this is particularly obvious inside of the Hymenoptera order. Among these residues, Y_406_ was found to be present in all VKRs (except in lepidopteran sequences). Y_406_ is exactly at the position K_509_ in mGluR1, the residue involved in the binding of the terminal carboxylic group of glutamate [12], and this strengthens our observation that glutamate is not a ligand for VKR. Additionally, we can see in the VFT alignment several conserved residues and motifs (highlighted in red, Figure 5), such as C_35_ and W_128_ which are present in all domains and the motif (G_247_Y(V/I)WFLPxWL_256_) which is present in most VKRs. Finally to investigate the potential importance of these conserved residue positions for the VFT properties, we performed a comparative modeling of the VFT domain of VKR using the ModWeb server of the ModBase databases. A VFT model of AmVKR was generated with the human Glutamate Receptor 5 (mGluR5) as homologous template (PDB: 3lmkA) (Figure 6). Results confirmed the position inside of the ligand pocket formed between the two lobes of the VFT, of the residues potentially involved in amino-acid recognition. The model also indicates that many of the other conserved residues are constituents of alpha-helices or beta-sheets, and thus take part very likely in the tertiary structure of the domain. Interestingly, two residues T_108_ and I_109_, highly conserved in most VKRs, are located in the putative ligand binding pocket, suggesting that they could contribute with the conserved residues of the amino-acid binding motif to ligand specificity and/or affinity. Taken together, these results let suppose that in spite of a partial conservation of primary structures, the VFT domains of many VKRs can bind a common ligand.

## Conclusions

This survey based on the analysis of newly released genomic data has allowed us to show that *vkr* genes actually represent a novel RTK family, widespread in the bilaterian branch of Eumetazoa. From this study we can extend the presence of VKR to a large variety of protostomes (Ecdysozoa and Lophotrochozoa). However, after the analysis of a large number of deuterostome genomes (Chordata and Hemichordata), it seems that deuterostomes would not contain *vkr* genes, with the exception of echinoderms. In these studies, an important information concerns the detection of NveVKR in the basal metazoan *N*. *vectensis*. From the presence of a *vkr* gene in Cnidaria, we can suggest that the origin of VKR would be anterior to the radiation of Bilateria, and possibly close in time to that of the setting-up of animal multicellularity. This would agree with the general acceptance that emergence of a series of cell surface receptors (including RTKs) necessary for cell adhesion, differentiation and cell-cell communications has driven evolution towards multicellularity [6]. In this context, we have recently found putative RTKs exhibiting an architecture close to that of VKR proteins in the genomes of the choanoflagellates *M*. *brevicollis*[5] and *S*. *rosetta*[17], which are free-living unicellular and colonial flagellates considered to be the closest living relatives of the animals. These findings (unpublished) encourage us to postulate that VKR could represent an ancient RTK present early in protists, that might have contributed to the establishment of multicellularity and to animal development.

Another important question concerns the distribution and stability of VKR throughout evolution. *Vkr* genes were found preferentially in the genomes of protostomes and particularly in insect genomes, but the large number of insect VKR sequences could likely result from the relative abundance of insect sequences in genomic databases. However, it was very interesting to observe that the finding of one *vkr* in all species of a given genus is not a general rule. For example, in the *Drosophila* genus, *D*. *melanogaster* and some others exceptionally do not possess a *vkr* gene [12]. Also, it is surprising that no *vkr* exists in other ecdysozoa like nematodes, and in *C*. *elegans* particularly.

In this work, we have analysed the exon/intron structure of all *vkr* genes and shown that their organization is widely heterogeneous across the different phyla. However, all *vkr* genes share intron positions common to the ancestral gene *Nvevkr*, and this suggests that they might have been derived from this common ancestor, then subjected to more or less marked reorganization along evolution. Finally, the question of the existence of two *vkr* genes in Trematoda and Lepidoptera is still open, together with the problem of the “keep or loss” of *vkr* in some species. Further investigations about the functions of VKR in the biology and physiology of organisms should be required to answer these questions.

We have previously shown that *vkr* genes were preferentially expressed in larval stages and in gonads of several organisms, including sea urchin, mosquito and the trematode *S*. *mansoni*, thus suggesting a role of the receptor in embryogenesis and gonad development [11,12,14]. About their functional activity, we have been able to demonstrate the tyrosine kinase activity of several VKRs [12,14] and sequence information here obtained for the TK domains of all VKRs confirms that most VKRs should be active kinases as well. VKR receptor activation was shown to be dependent on the binding of L-amino-acids, and specifically of L-arginine, to the extracellular VFT domain, in which conserved residue positions (for example S_66_ and R_107_) are essential for ligand-receptor interaction and probably involved in specificity and affinity of L-arginine. However, in some VKRs, these major conserved residues are not present, suggesting that they could bind other ligands and perhaps have different functional activities. We have shown that SmVKR1 and SmVKR2 of *S*. *mansoni* differ both in the primary sequence of their VFT domain and in their localization in the parasite. The observation that these receptors are activated respectively by L-arginine and Calcium [14] really illustrates this possible functional divergence between VKR.

Currently, diverse strategies are developed to analyse the consequences of VKR knock-down in organisms. Preliminary results of VKR targeting by RNA interference in *S*. *mansoni* have confirmed the importance of SmVKR1 and SmVKR2 for growth and differentiation of reproductive organs and parasite fertility (to be published). The use of other organisms as candidate models for studying the function of VKR is under investigation.

In conclusion, the VKR family is a little-known RTK family that deserves to be further explored in order to determine more precisely its evolutionary origin, its possible importance for the emergence and specialization of Metazoa, and to understand how its maintenance or its loss in various phyla or species could be in relation with development and physiological activities (like reproduction).

## Methods

### Genome database searches

Putative *vkr* sequences were searched using tBLASTn on genomic sequences available in the following databases: GenBank (http://www.ncbi.nlm.nih.gov/blast/Blast.cgi), FlyBase (http://flybase.org/), VectorBase (http://www.vectorbase.org), Wellcome Trust Sanger institute databases (http://www.sanger.ac.uk/resources/databases/) and the JGI genome portal (http://genome.jgi-psf.org/). Additionally, we also searched for *vkr* sequences in *Hydra magnipapillata* (*hydrazome*), *Schmidtea mediterranea* (*SmedDB*) and *Schistosoma japonicum*(*schistoDB*) genome databases.

Selected genomic sequences were analysed by GenScan (http://genes.mit.edu/GENSCAN.html) and Augustus (http://bioinf.uni-greifswald.de/augustus/) gene prediction servers, and putative VKR proteins were then determined. The presence of VFT, TM and TK domains was verified, and their delimitation defined using the SMART software (http://smart.embl-heidelberg.de/). Finally, TM regions were confirmed with the TMHMM server (http://www.cbs.dtu.dk/services/TMHMM).

### Phylogenetic analyses

Protein sequences (listed in Additional file 1) were aligned using ClustalW algorithm in the BioEdit v7.1 software, and manually corrected. Maximum likelihood trees were built using MEGA5 [25] under the JTT+I+G model, with 100 bootstrap repetitions.

### Comparative modeling

Comparative modeling was performed on the VFT sequence domain of VKRs using the comparative modeling web-server Modweb (https://modbase.compbio.ucsf.edu/scgi/modweb.cgi) of the ModBase databases. Calculation and evaluation of models were performed with ModPipe software using sequence-sequence, sequence-profile and profile-sequence methods for fold assignment and target-template alignment.

## Competing interests

The authors declare that they have no competing interests.

## Authors’ contributions

MV, NG and AA carried out research of genomic sequences in databases and sequence analysis using gene prediction servers. MV and NG performed functional annotation based on sequence similarity using SMART and sequence alignments of protein sequences. MV and MM performed phylogenetic studies. NG made comparative modeling experiments. JV and CD participated in the design of the experiments. CD coordinated the study. MV and CD wrote the manuscript. All the authors read and approved the final manuscript.

## Supplementary Material

Additional file 1 **FastA text file containing the full length sequences of the 40 VKR proteins aligned using the Clustal W algorithm.** For VKR abbreviations, see Table 1. Click here for file
